# Green synthesis of nanohydroxyapatite trough *Elaeagnus angustifolia L.* extract and evaluating its anti-tumor properties in MCF7 breast cancer cell line

**DOI:** 10.1186/s12906-023-04116-3

**Published:** 2023-09-26

**Authors:** Asghar Zarban, Ehsaneh Azaryan, Maryam Moradi Binabaj, Samira Karbasi, Mohsen Naseri

**Affiliations:** 1 Cardiovascular Diseases Research Center, Birjand University of Medical Sciences, Birjand, Iran; 2 Clinical Biochemistry Department, Faculty of Medicine, Birjand University of Medical Sciences, Birjand, Iran; 3 Cellular and Molecular Research Center, Department of Molecular Medicine, Birjand University of Medical Sciences, Birjand, Iran; 4 Cellular and Molecular Research Center, Sabzevar University of Medical Sciences, Sabzevar, Iran

**Keywords:** MCF-7 breast cancer, *Elaeagnus Angustifolia*, Hydroxyapatite nanoparticle, Apoptosis

## Abstract

**Background:**

One of the most common types of cancer in women is breast cancer. There are numerous natural plant-based products, which exert anti-tumoral effects including *Elaeagnus Angustifolia* (EA). It modulates cell-cycle process, heat-shock proteins expression, anti-proliferative properties, apoptosis induction, blocking of angiogenesis, and cell invasion inhibition. The current study aimed to synthesize and evaluate the anticancer effects of hydroalcoholic EA extract (HEAE), Nanohydroxyapatite (nHAp) and nHAp synthesized trough EA (nHA-EA) in MCF-7 breast cancer cell line.

**Methods:**

In the present study, HEAE preparation and green synthesis of nHA-EA was done and phase composition, functional groups, and crystallin phase of nHA-EA and nHAp were determined using Fourier-transform infrared (FTIR) and X-ray diffraction (XRD). The characteristics of synthesized nanoparticles including structural and morphological parameters were investigated using scanning electron microscopy (SEM) and Transmission electron microscopy (TEM) techniques. Then, by using MTT-assay (Dimethylthiazoldiphenyltetrazolium), the in vitro cytotoxic and half maximal inhibitory concentration (IC_50_) of EA extract, nHAp, and nHA-EA in the MCF-7 breast cancer cell line was evaluated. Next, we assessed the expression of apoptosis-related genes Bax, Bcl2 and p53 using quantitative reverse-transcriptase polymerase-chain-reaction (qRT-PCR) and migration of MCF-7 cells by scratch assay.

**Results:**

The FTIR results demonstrated formation of nHAp and its interaction with HEAE during synthesis process. The XRD results of the synthesized nanoparticles showed similar XRD pattern of nHA-EA and nHAp and purity of synthesized nanomaterials. The average IC_50_ of HEAE, nHAp, and nHA-EA extract after treatment of cancer cells for 24 h was 400 µg/mL, 200 µg/mL, and 100 µg/mL, respectively. Our results revealed that nHA-EA significantly reduced the migration and invasion of the MCF-7 cells, in comparison to the nHAp and EA extract. Moreover, level of Bax/Bcl2 and p53 was significantly higher in the nHA-EA extract group in comparison to the EA extract and nHAp group.

**Conclusion:**

Taken together, our results demonstrated that bioactive constituents of EA medicinal plant in form of nHA-EA particles, can effectively exerts potential anticancer and chemo preventive effect against breast cancer growth and can be proposed as a promising beneficial candidate for BC therapy. However, further investigations are required to discover what bioactive compounds are responsible for the chemo preventive effect of this extract.

## Introduction

One of the most common types of cancer in women is breast cancer (BC) [1], and it is considered a leading cause of death in patients with cancer all around the world [2]. Despite the recent advances in BC diagnostic and therapeutic methods, due to the high rate of mortality and chemoresistance, BC cancer patients' treatment is still a matter of debate [3].

There are numerous natural plant-based products and extracts, which exert anti-tumoral effects and could be useful for the design and development of new anti-cancer drugs [4]. *Elaeagnus Angustifolia* (EA) (also called oleaster, Russian olive, wild olive, silver berry), is a tree, that belongs to the Elaeagnacea (Araliaceae) family, and its fruits are characterized by small size and reddish-brown color. Different type of EA is commonly growing in Asia, Europe, and some regions of North America [5, 6]. The fruit of EA has been utilized as a medicinal plant, which acts as a potential therapeutic agent in numerous disorders through modulation of the immune system and oxidative stress balance [5]. It also exerts anti-cancer, antibacterial, and antifungal effects and has gastro- and hepatoprotective efficacy [7–10]. It has been shown that EA fruit is a rich source of different vitamins, carbohydrates, proteins, and minerals [6, 11, 12]. Moreover, EA fruits are a good source of beneficial compounds such as coumarins, tannins, phenolic acids, and flavonoids [5, 13, 14]. Different studies are showing that EA exerts anticancer effects via modulating the cell-cycle process, heat-shock proteins expression, anti-proliferative properties, apoptosis induction, blocking of angiogenesis, and cell invasion inhibition [15–17].

Nanomedicine has drawn a lot of interest because of its various and effective utilization in medicine, particularly in drug delivery [18]. Due to the high dispersion stability of nanoparticles (NPs), they have attracted considerable research interest in biomedical applications and drug delivery systems [19, 20]. The biosynthesis of NPs, using natural sources such as plants, without the utilization of any hazardous substances, reduces potential health and environmental threats [21, 22]. Moreover, plant extract allows the preparation of NPs with controlled and defined shape and size [23].

Hydroxyapatite (HAp, Ca_10_(PO4)_6_(OH)_2_), has various biomedical applications in many areas of medicine, due to its high bioactivity and biocompatibility. According to the method applied for HAp preparation, various mechanical properties bioactivity level, and dissolution behavior in the biological environment is expected [24]. It has been shown that HAp exerts anti-cancer effects via increasing drug release and consequently greater growth inhibition properties. Taken together, HAp enhances the chemotherapeutic efficacy of various agents including Cisplatin, Methotrexate, and Adriamycin. This could be because HAp mediates drug penetration into the tumor and improves drug delivery [25] improvement. It has been shown that the green synthesis of NPs has opened up new possibilities in material development, and there is research conducted on the green synthesis of HAp [26].

A different source of flavonoids including, catechin, epicatechin, gallocatechin, epigallocatechin, kaempferol, quercetin, luteolin, isorhamnetin, and isorhamnetin-3–0-β-D-galactopyranoside have been isolated from EA [27]. Furthermore, various phenolic components such as 4- hydroxybenzoic acid and caffeic acid have also been found in EA [28]. It has been shown that flavonoids contain phenolic hydroxyl groups, they may play a role in metals chelating efficiency, lowering lipid peroxidation process, and increasing antioxidant and free radical scavenging capacity [29, 30]. According to these findings, in the current study, nano hydroxyapatite (nHAp) was synthesized with the EA extract, which shows reducing/stabilizing effects, as well as capping properties. This synthesis method is inexpensive and simple, has long-lasting stability, and is appropriate for macro-scale procedures [7, 31–33].

Therefore, the current study aimed to synthesize and evaluate the anticancer effects of hydroalcoholic EA extract (HEAE), nHAp, and nHAp synthesized trough EA (nHA-EA) in MCF-7 breast cancer cell line. The synthesized nanoparticles were characterized, and their structural, morphological, and optical properties were determined via different analytical tools, including x-ray diffraction (XRD), fourier-transform infrared (FT-IR), scanning electron microscopy (SEM), and transmission electron microscopy (TEM).

## Materials & methods

In the present study, all chemicals and reagents used to synthesize nHAp and nHA-EA were of analytical grade. Calcium nitrate tetrahydrate [Ca (NO3)2·4H2O], diammonium hydrogen phosphate [(NH4)2 HPO4], sodium hydroxide (NaOH), and were purchased from Sigma Aldrich. All chemicals solution was prepared using deionized water.

### Plant material and extract preparation

The Russian Olive was obtained from South Khorasan, Birjand, Iran. It was identified as *Elaeagnus Angustifolia L*. by Dr. F. Askari (Assistant Professor of Traditional Pharmacy, School of Pharmacy). The voucher specimen was deposited in the Herbarium Center of the School of Pharmacy, Birjand University of Medical Sciences (221).

### Hydroalcoholic EA extract preparation & green synthesis of nHA-EA

We have discussed synthesis of nHAp and nHA-EA in the previous studies [29, 34]. Briefly, EA pulps were obtained from fresh fruits and HEAE was prepared using the maceration procedure as below. 40 gr of dried EA pulp powder with 320 mL methanol and 80 ml distilled water were mixed. Finally, the extracts were filtered and concentrated using a rotating vacuum and HEAE was stored at 4 °C.

**Fig. 1:**
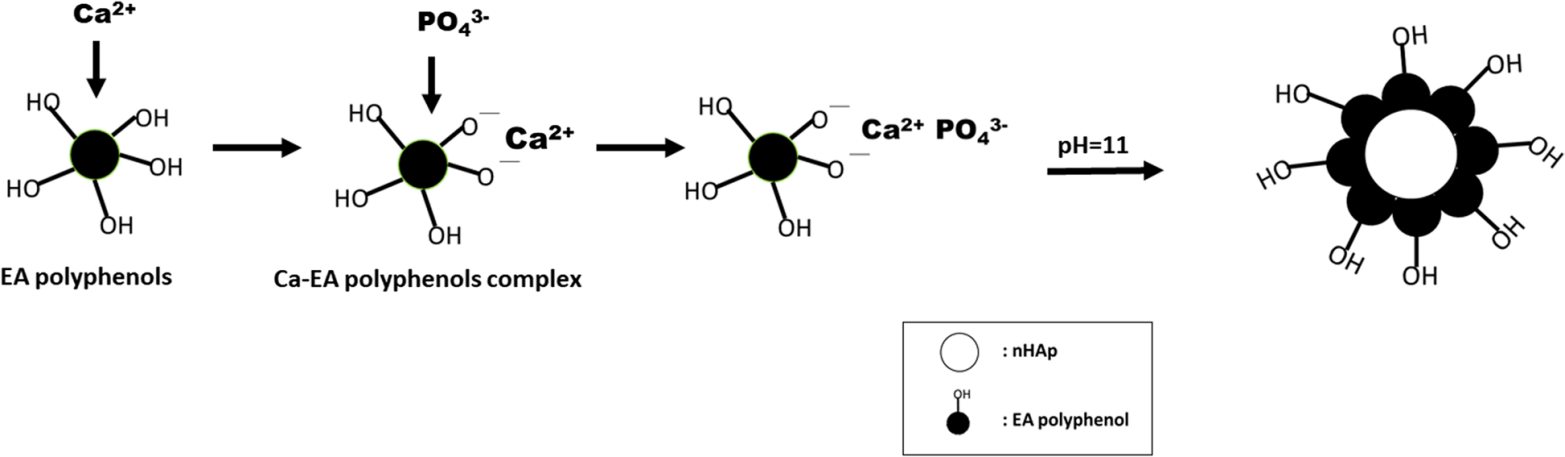
A schematic representation of the nHA-EA preparation process

nHA-EAs were synthesized using the sol–gel technique, using calcium nitrate tetrahydrate and diammonium hydrogen phosphate, (molar ratio: 1.67). V/V HEAE (10 ml, 10%), diammonium hydrogen phosphate (5 ml, 0.3 M), and calcium nitrate tetrahydrate (15 ml, 0.3 M) were dissolved in ionized water. The Calcium nitrate tetrahydrate solution was introduced to the HEAE solution, then stirred upon slow warming to 50 °C, for 0.5 h. Then, diammonium hydrogen phosphate solution was added to the above-mixed solution at the flow rate of 1 ml/min. NaOH solution was used for pH adjustment (pH = 11). At 50 °C for 90 min, the suspension was agitated (Fig. 1). The resulting solution was subjected to the centrifuge and then gently rinsed with deionized water and ethanol (nHA-EA). Finally, nHAp solution without HEAE was synthesized under the same situations described for comparison.

### Characterization

The phase composition, functional groups, and crystallin phase of nHA-EA and nHAp were determined using FTIR and XRD. The characteristics of synthesized nanoparticles including structural and morphological parameters were investigated using SEM and TEM techniques.

### Cell culture

The human MCF-7 cells were purchased from the Pasteur institute (Tehran, Iran). The cells were cultured in dulbecco's modified eagle's medium (DMEM) supplemented with 10% heat-inactivated fetal bovine serum (FBS) and 1% streptomycin/ penicillin and maintained at 37 °C in a 5% CO2 atmosphere.

### MTT-assay

The MTT-assay (Dimethylthiazoldiphenyltetrazolium) was used to assess the in vitro cytotoxic and half maximal inhibitory concentration (IC_50_) of EA extract, nHAp, and nHA-EA in the MCF-7 breast cancer cell line. Cells were treated with different concentrations of EA extract, nHAp, and nHA-EA for 24 h. Then, the medium was removed from the wells and MTT substrate (Sigma, Germany) was added to each well, and the plate was incubated for 4 h. Then, the resulting crystals were dissolved in dimethylsulfoxide (DMSO) and incubated for 1 h. Finally, the absorbance was measured at 540 nm using a microplate reader. The experiments were performed in triplicate.

### Scratch assay

Briefly, MCF-7 cells were seeded in a 12-well plate at a concentration of 4 × 10^5^ cells /well. Then, a narrow scratch was created with a 100 μl sterile pipette tip through a monolayer of adherent cells growing on the bottom of a cell culture plate, 24 h after cell seeding. Then, cell debris was removed. Plates were treated and after incubation at different time points were imaged and analyzed by using Digimizer 5.4.9 software.

### Quantitative reverse-transcriptase polymerase-chain-reaction (qRT-PCR)

In this study, a quantitative RT-PCR assay was used to measure transcript levels of different genes. First, total RNA was extracted from the cells using the Pars Tous kit, (Tehran, Iran) according to the manufacturer's instructions. Then, RNAs were converted to complementary DNA (cDNA) using the commercial cDNA synthesis kit (Parstous kit). Quantitative RT-PCR was performed with specific forward and reverse primers for target genes (Table 1). The cDNA amplification was performed by using the StepOne instrument (Applied Biosystems, Foster City, CA). The gene expression levels were normalized to a housekeeping control gene, glyceraldehyde-3-phosphate dehydrogenase (GAPDH).

**Table 1:** Primers used for real-time polymerase chain reaction

Gene Symbol	Gene Name	Forward Primer	Reverse Primer
**GAPDH**	Glyceraldehyde-3-phosphate Dehydrogenase	TCAAGATCATCAGCAATGCCTCC	GCCATCACGCCACAGTTTC
**Bax**	Bcl-2-associated X protein	TGACGGCAACTTCAACTGGG	CTTCAGTGACTCGGCCAGGG
**Bcl2**	B-cell lymphoma 2	GTCATGTGTGTGGAGAGCGTC	CCGTACAGTTCCACAAAGGCATC
**p53**	Tumor suppressor protein	ACACGCTTCCCTGGATTGG	CTAGGATCTGACTGCGGCTC

### Statistical analysis

All values are expressed as mean ± standard error of the mean. GraphPad Prism (version 9) was used to conduct the analyses. Statistical comparisons were determined using student’s t-test or one‐way analysis of variance (ANOVA) followed by tukey’s multiple comparison test. The differences were considered to be statistically significant at *P* < 0.05.

## Results

### FTIR

**Fig. 2:**
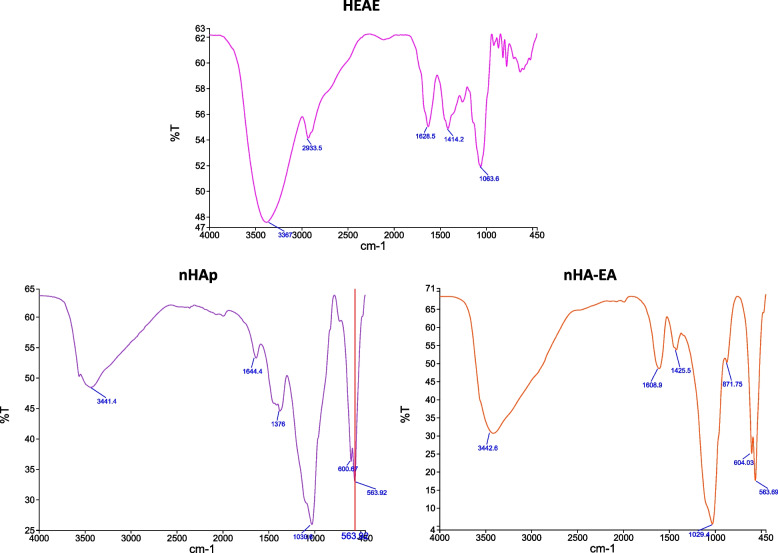
FTIR spectra of HEAE, nHAp and nHA-EA

The FTIR technique in the middle infrared spectral (400–4000 cm^−1^) wavelength was used to evaluate the functional groups of HEAE, nHAp and nHA-EA (Fig. 2). In the HEAE spectra, the continued peak at 3367 cm^−1^ is defined to O–H vibration and peaks at 1060–1283 cm^−1^ are the C–O–C characteristic peaks. In the nHAp, the intense peak around at 564–601 and 1030 cm^−1^ indicated the PO4^3−^ bands. Moreover, the O–H vibrations on the water molecule were found around 3000 to 3600 cm^−1^. In the nHA-EA spectra, the extended peak around at 3443 shows water absorption in the synthesized nanoparticles. nHA-EA spectra analysis showed distinct bands of PO4^3−^ at 564, 604, and, 1029 cm^−1^. The extra peak at 870 showed the presence of C = O stretching of carboxylic acid, and clearly suggests the interaction between carboxyl group and nHAp. These FTIR functional groups findings clearly demonstrates formation of nHAp and its interaction with HEAE during synthesis process.

### XRD

**Fig. 3:**
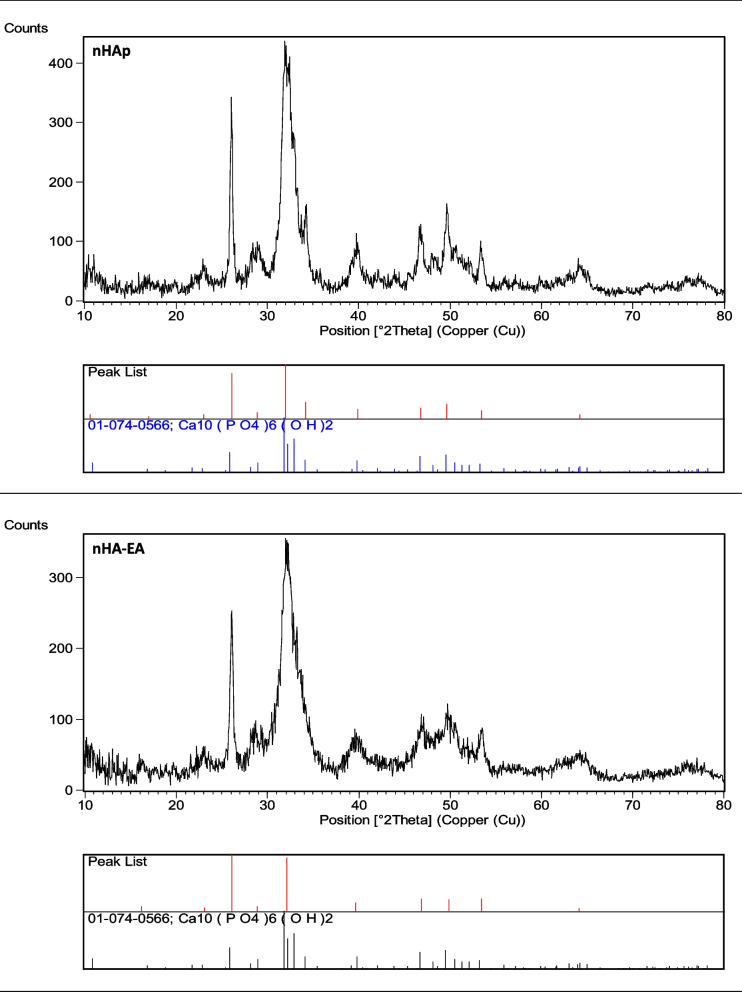
XRD patterns of nHA and nHAEA

The X-ray diffraction patterns results of the synthesized nanoparticles are presented at Fig. 3, nHA-EA and nHAp showed similar XRD pattern. There was no peak related to the calcium hydroxide and calcium phosphates, which indicates synthesized nanomaterials purity. The seven peaks observed at 26◦, 32◦, 39◦, 46◦, 49◦,53◦ and 64◦, were corresponded to the plane (002), (112), (310), (222), (123), (004), and (233), respectively, and related to the hydroxyapatite, which find to be matched with ICDD: 01–084-1998.

### SEM and TEM

**Fig. 4:**
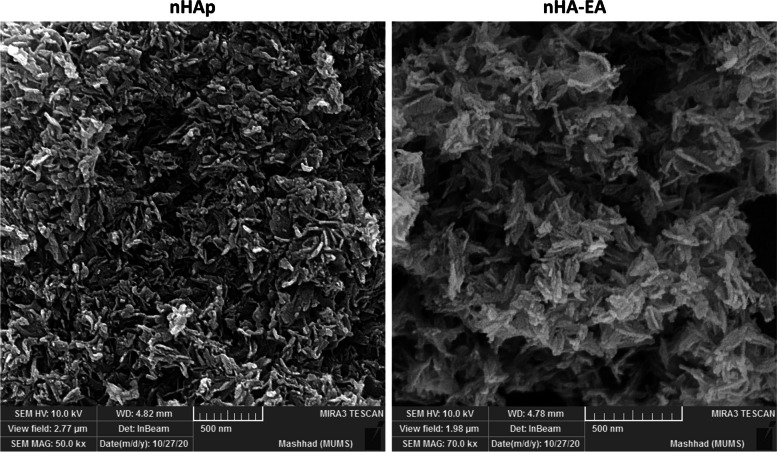
SEM images of nHAp and nHA-EA

**Fig. 5:**
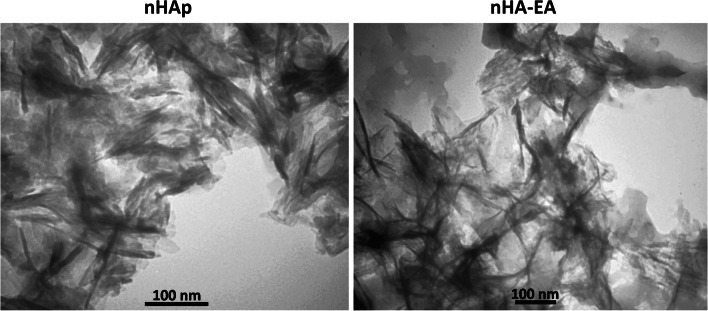
TEM images of nHAp and nHA-EA

Data obtained from SEM and TEM images revealed nanorod particles (Figs. 4 and 5). Notably, nHAps have 17–29 nm width and 62–89 nm length, whereas nHA-EA nanorods have 17–23 nm width and 93–146 nm length.

### Cell proliferation

**Fig. 6:**
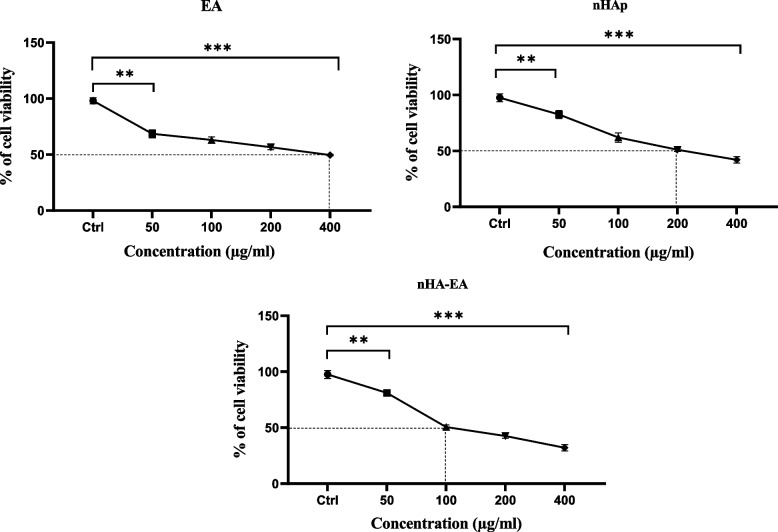
MTT assay images show cytotoxic effects and IC_50_ values of EA, nHAp and nHA-EA

In the current study, an MTT assay was performed to evaluate the in vitro cytotoxic and IC_50_ of EA, nHAp, and nHAEA extract in the MCF-7 breast cancer cell line. Our results indicated that the average IC_50_ of EA, nHAp, and nHAEA extract after treatment of cancer cells for 24 h was 400 µg/mL, 200 µg/mL, and 100 µg/mL, respectively (Fig. 6).

### Scratch assay

**Fig. 7:**
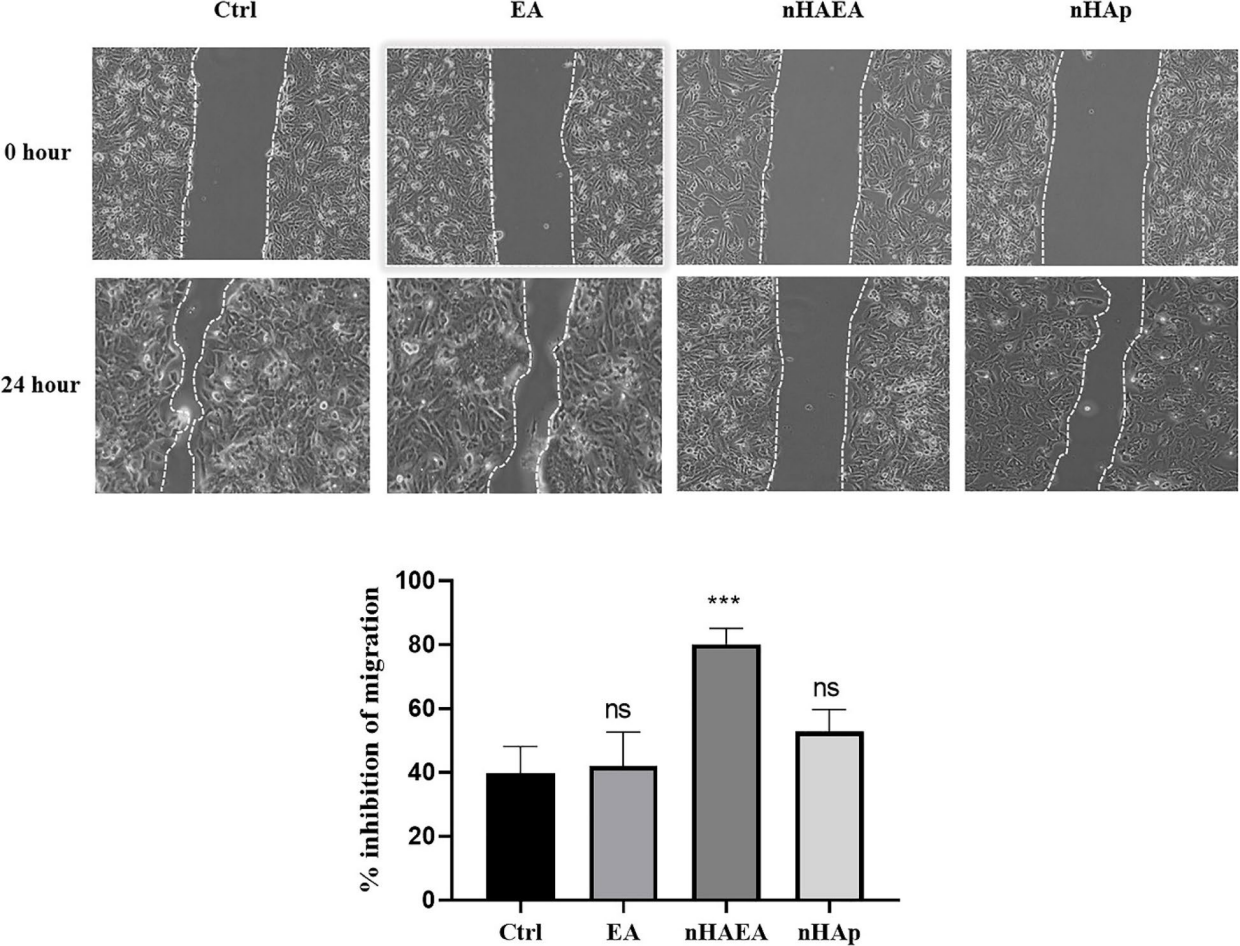
The effects of EA, nHAp and nHA-EA on the migration of the MCF-7 cells after 24 h

Regarding the potential effects of EA nanoparticles on breast cancer cell migration, in the present study, we examined the breast cancer cells invasiveness inhibitory effects of nHA-EA using the scratch assay. Our results revealed that nHA-EA significantly reduced the migration and invasion of the MCF-7 cells, in comparison to the nHAp and EA extract (Fig. 7).

### qRT-PCR

**Fig. 8:**
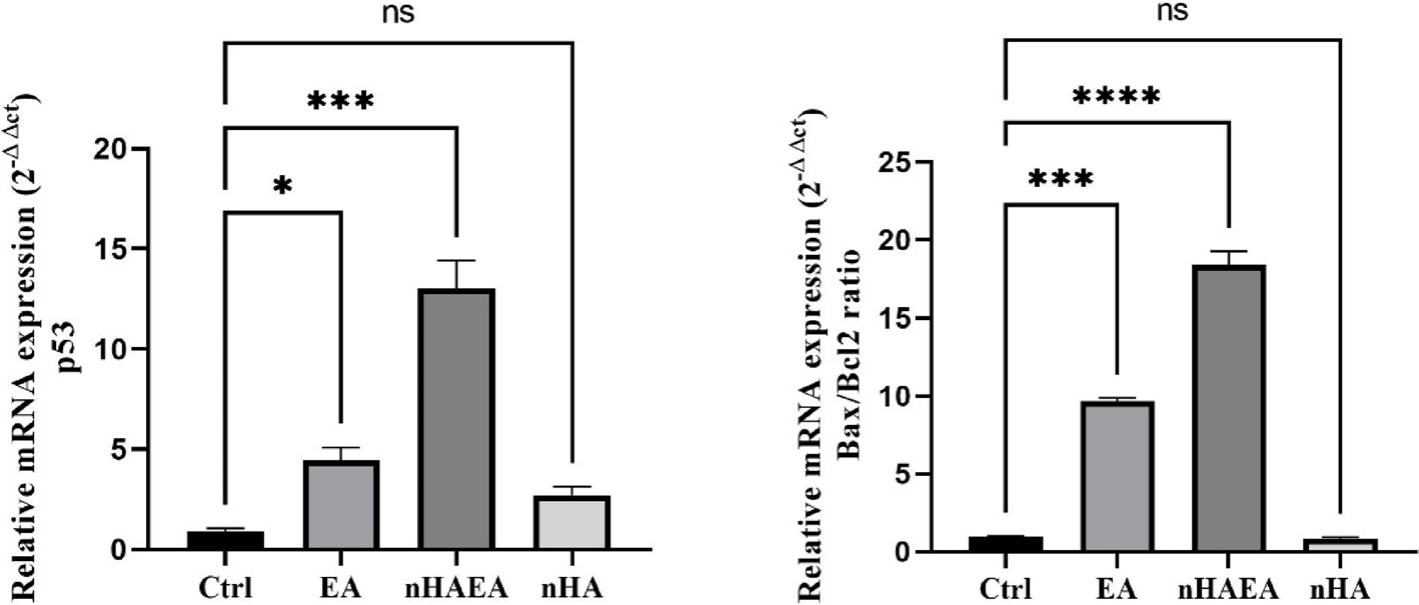
EA, nHAp and nHA-EA triggered MCF-7 cells apoptosis genes as showed by qRT-PCR

To determine the anti-cancer effects of EA extract, nHAp, and a combination of EA extract and nHAp in the MCF-7 cells, we evaluated the expression level of Bax/Bcl2 and p53 as key markers in the carcinogenesis process. Our results revealed that the level of Bax/Bcl2 and p53 are significantly higher in the nHA-EA extract group in comparison to the EA extract and nHAp alone (Fig. 8).

## Discussion

In the past few years, there have been reports of nanoscale HAp that exhibit superior biocompatibility compared to regular HAp. Furthermore, these nanoscale HAp particles have shown anti-cancer properties and play a crucial role in controlling the behavior of breast cancer cells [35]. There are multiple methods for synthesizing nHAp, such as co-precipitation, hydrothermal, sol–gel, and others. Additionally, the utilization of metal nanoparticles extracted from plants is considered environmentally friendly. As a result, the use of natural materials in the production of nHAp has captured the interest of numerous researchers, contributing to the development of a significant field of study within the realm of nanotechnology science [29].

A wide range of natural plant-derived products and extracts possess anti-tumoral properties, presenting potential value in the creation and advancement of novel anti-cancer nanoparticles. There are growing body of evidences showing that natural products have an important role in treatment of wide variety of human disease, including types of cancer [30, 36]. EA, as an herbal medicine plant with different properties, have been used extensively for a long time to treat different disorders [13, 33]. There are various bioactive constituent in EA, including phenolic acids and flavonoids, which exerts a critical role in cancer development and progression inhibition [13, 27]. It has been shown that bioactive compounds of EA could modulate different biological processes of cells including cell cycle progression, apoptosis and DNA repair [28, 37]. In recent years, EA has received considerable attention for cancer therapy because of its promising effectiveness and potential therapeutic effects as chemo preventive and antitumor natural product. It has been shown that flavonoids with different signaling pathways which are correlated with carcinogenesis, including cellular proliferation, apoptosis, angiogenesis, and metastasis. Moreover, apigenin, as a phytoestrogen aglycone has shown to suppress apoptosis, cell cycle and invasion in malignant cells, alone or in combination with other chemotherapeutic agents [38].

In this study, nHAp was synthesized with EA extract, which could act as a reducing, stabilizing, and capping agent. This synthesis is easy, cheap, durable, and suitable for largescale processing. To determine the anticancer effects of nHA-EA in MCF-7 cells were treated with nHA-EA.

According to result of this study, nHA-EA had more growth inhibitory properties on MCF-7 breast cancer cell line than other groups. Our results revealed that, nHA-EA decreased the viability and proliferation of MCF-7 cells and significantly increased the expression level of p53 and Bax/Bcl2 genes.

Flavonoids have been demonstrated to increase the expression of p53 and induce cell cycle arrest specifically in the G2/M phase in cancer cells. Additionally, they are recognized for their ability to inhibit the expression of Ras proteins and modulate heat-shock proteins in different types of cancers, particularly in leukemia and colorectal cancer.

The flavonoids in the EA extract surround the nHAp like a cap. As a result, they may increase the anti-cancerous properties of nHAp. Quercetin, a prominent flavonoid found in EA, is a significant anti-proliferative compound. In addition, it plays a role in enhancing tumor necrosis factor-related apoptosis-inducing ligand (TRAIL) by increasing the expression of Bax and suppressing the activity of Bcl2 protein [39].

These results are in accordance with previous studies showing the suppressive effects of EA on different type of cancer including glioblastoma and breast cancer [38, 40]. Although, the results of the current study are not consistent with other investigation, showing that EA extract did not exerted any significant antiproliferative activity against cancerous cells, which may be due to the different concentration of extract and its characteristics [41].

Uncontrolled cell division and growth and escape from apoptosis are well-known hallmarks of malignant cells and important mechanism involved in cancer treatment resistance; therefore, targeting specific cancer deregulated pathways could be an important way for providing novel therapeutics strategies [42, 43].

Moreover, we revealed that the ethanolic extract of EA could decrease migration of MCF-7 cells and induces apoptosis in this cell line. Our results are in agreement with other studies that confirmed EA exerts its anti-tumor impacts via upregulation of p53 and Bax/Bcl2.

The observed anti-proliferative effects of EA extract on breast cancer cells in our study could mediate via increasing the expression of p53 as a tumor suppressor gene. It has been shown that bioactive constituents of in EA, including flavonoids, triterpenoid, lignanoid, benzenoid, pro-anthocyanosides, polysaccharides and phenolic acids, plays an important role in preventing cancer development and progression. Investigations revealed that these constituents could regulate various biological pathways including apoptosis, DNA repair, inflammatory processes and cell cycle progression [13, 33, 44]. p53 regulates the expression of Bcl2, a proapoptotic factor, in which triggers apoptosis process [45]. Moreover, there are studies showing that EA extract induces apoptosis via different mechanisms including, human epidermal growth factor receptor 2 gene (HER2) and Jun N-terminal kinases (JNK) inhibition [39]. Thus, these results support the role for EA extract in the Bcl2-associated intrinsic pathway of apoptosis via p53 upregulation.

It is well known that invasion and migration, which are key underling mechanisms of metastasis plays an important role in breast cancer disease. Metastasis is a process in which malignant cells leaving primary tissue, disseminate, circulate and induces secondary tumors in distant sites [46].

In the present study, analysis of scratch assay revealed that, nHA-EA significantly inhibited migration and invasion in MCF-7 cell line. The anti-migratory and anti-metastatic potential of EA herbal plant has been confirmed in a study conducted by Jabeen et.al. showing that, EA extract inhibited the invasiveness of HER2-positive breast cancer cell lines. They showed that, this effect is associated with JNK signaling suppression and mesenchymal-epithelial transition (MET) inhibition. They also reported that suppression of JNK signaling pathway is associated with induction of apoptosis process [39]. In another study conducted by Saleh et.al, it has been found that EA extract suppresses the invasiveness and migration of oral carcinoma cell lines. They reported that this extract inhibits cell invasion via increasing the amplification of E-cadherin, an important modulator of MET process. The also indicated that this reduction in migratory properties of oral carcinoma cell lines is mediated via inhibition of extracellular-signal-regulated kinases 1 and 2 (ERK1/ERK2) pathway [47].

## Conclusion

Taken together, our results demonstrated that bioactive constituents of EA medicinal plant in form of nHA-EA particles, can effectively exerts potential anticancer and chemo preventive effect against breast cancer growth and can be proposed as a promising beneficial candidate for BC therapy. Moreover, we showed that nHA-EA triggers cell apoptosis in MCF-7 cells via increasing proapoptotic gene. Additionally, our findings indicate the role of nHA-EA as a natural product in targeting p53 as a key marker in BC treatment. However, further investigations are required to discover what bioactive compounds are responsible for the chemo preventive effect of this extract.

## Data Availability

The datasets used and/or analysed during the current study available from the corresponding author on reasonable request.

## Abbreviations

EA: Elaeagnus Angustifolia
HEAE: Hydroalcoholic EA extract
nHAp: Nano hydroxyapatite
nHA-EA: nHAp synthesized trough EA
FTIR: Fourier-transform infrared
XRD: X-ray diffraction
SEM: Scanning electron microscopy
TEM: Transmission electron microscopy
BC: Breast cancer
NPs: Nanoparticles
Ca (NO3)2·4H2O: Calcium nitrate tetrahydrate
(NH4)2HPO4: Diammonium hydrogen phosphate
MTT: Dimethylthiazoldiphenyltetrazolium
cDNA: Converted to complementary DNA
GAPDH: Glyceraldehyde-3-phosphate dehydrogenase
ANOVA: One‐way analysis of variance
DMEM: Dulbecco's Modified Eagle's Medium
qRT-PCR: quantitative reverse-transcriptase polymerase-chain-reaction
DMSO: Dimethylsulfoxide
FBS: Fetal bovine serum
MET: Mesenchymal-epithelial transition
HER2: Human epidermal growth factor receptor 2 gene
JNK: Jun N-terminal kinases
ERK1/ERK2: Extracellular-signal-regulated kinases 1 and 2
TRAIL: tumor necrosis factor-related apoptosis-inducing ligand
IC50: Half maximal inhibitory concentration
